# Comparison and Fusion of Deep Learning and Radiomics Features of Ground-Glass Nodules to Predict the Invasiveness Risk of Stage-I Lung Adenocarcinomas in CT Scan

**DOI:** 10.3389/fonc.2020.00418

**Published:** 2020-03-31

**Authors:** Xianwu Xia, Jing Gong, Wen Hao, Ting Yang, Yeqing Lin, Shengping Wang, Weijun Peng

**Affiliations:** ^1^Department of Radiology, Municipal Hospital Affiliated to Medical School of Taizhou University, Taizhou, China; ^2^Department of Radiology, Fudan University Shanghai Cancer Center, Shanghai, China; ^3^Department of Oncology, Shanghai Medical College, Fudan University, Shanghai, China

**Keywords:** lung adenocarcinoma, deep learning, radiomics, invasiveness risk, ground-glass nodule, CT scan

## Abstract

For stage-I lung adenocarcinoma, the 5-years disease-free survival (DFS) rates of non-invasive adenocarcinoma (non-IA) is different with invasive adenocarcinoma (IA). This study aims to develop CT image based artificial intelligence (AI) schemes to classify between non-IA and IA nodules, and incorporate deep learning (DL) and radiomics features to improve the classification performance. We collect 373 surgical pathological confirmed ground-glass nodules (GGNs) from 323 patients in two centers. It involves 205 non-IA (including 107 adenocarcinoma *in situ* and 98 minimally invasive adenocarcinoma), and 168 IA. We first propose a recurrent residual convolutional neural network based on U-Net to segment the GGNs. Then, we build two schemes to classify between non-IA and IA namely, DL scheme and radiomics scheme, respectively. Third, to improve the classification performance, we fuse the prediction scores of two schemes by applying an information fusion method. Finally, we conduct an observer study to compare our scheme performance with two radiologists by testing on an independent dataset. Comparing with DL scheme and radiomics scheme (the area under a receiver operating characteristic curve (AUC): 0.83 ± 0.05, 0.87 ± 0.04), our new fusion scheme (AUC: 0.90 ± 0.03) significant improves the risk classification performance (*p* < 0.05). In a comparison with two radiologists, our new model yields higher accuracy of 80.3%. The kappa value for inter-radiologist agreement is 0.6. It demonstrates that applying AI method is an effective way to improve the invasiveness risk prediction performance of GGNs. In future, fusion of DL and radiomics features may have a potential to handle the classification task with limited dataset in medical imaging.

## Introduction

As the most common histologic subtype of lung cancer, lung adenocarcinomas accounts for almost half of lung cancers. The persistent presence of ground-glass nodules (GGN) in computed tomography (CT) image usually serves as an indicator of the presence of lung adenocarcinoma or its precursors (1). According to the guideline of the 2011 International Association for the Study of Lung Cancer/American Thoracic Society/European Respiratory Society International (IASLC/ATS/ERS) classification, lung adenocarcinoma includes atypical adenomatous hyperplasia (AAH), adenocarcinoma *in situ* (AIS), and minimally invasive adenocarcinoma (MIA) and invasive adenocarcinoma (IA) (2). Previous reported studies has depicted that the different subtypes of lung adenocarcinoma have different 3-years and 5-years disease-free survival (DFS) rates (3). For stage-I lung adenocarcinoma, the 5-years DFS of AIS and MIA is 100%, but IA is only 38–86% (4, 5). Meanwhile, the standard surgical treatment for lung adenocarcinoma is still lobectomy, but non-IA patients may be candidates for limited surgical resection (6). Thus, it is important to discriminate between IA and non-IA (including AIS and MIA) by using non-invasive CT image.

In order to classify between non-IA and IA GGNs, investigators and researchers have proposed two kinds of computer-aided diagnosis (CADx) schemes including CT radiomics feature analysis method and deep learning (DL) architecture based scheme (7). The radiomics feature analysis approach mainly includes tumor segmentation, radiomics feature extraction and selection (8), and machine-learning classifier training/testing process, respectively (9–11). The related studies usually compute a large number of handcrafted imaging features to decode the different tumor phenotypes (6, 12–14). Unlike radiomics feature analysis scheme, DL based scheme use the convolutional neural network (CNN) to build an end-to-end classification model by learning a hierarchy of internal representations (15–17). Although DL scheme can improve the classification performance and reduce the workload of hand-craft feature engineering (i.e., tumor boundary delimitation), it needs to be trained with larger dataset than radiomics feature based scheme (18, 19). However, under common medical diagnosis conditions, collecting, and building a large uniform image dataset is very difficult because of the inconformity of CT screening standard and lacking surgical pathological confirmed GGNs. Thus, how to improve the CADx performance with a limited dataset is a challenge task.

To address this issue, we have fused the DL and radiomics features to build a new AI scheme to classify between non-IA and IA GGNs. We first collected 373 surgical pathological confirmed GGNs from 323 patients in two centers. To segment the GGNs in CT images, we trained a recurrent residual convolutional neural network (RRCNN) based on U-Net model. Then, we respectively built a DL model and radiomics feature analysis mode to classify between IA and non-IA GGNs. Finally, we applied an information fusion method to fuse the prediction scores generated by the two models. In order to evaluate the performance of our new scheme, we used an independent dataset to conduct an observer study by comparing our prediction score with two radiologists (an experienced senior radiologist S.P. Wang and a junior radiologist W. Hao).

## Materials and Methods

### Image Dataset

In this study, we respectively collected 373 surgical pathological confirmed GGNs from two centers. For the cases with multifocal ground-glass nodules (multi-GGNs), we treated each GGN as an independent primary lesion (20). The inclusion criteria were: (1) diagnosed with stage-I lung adenocarcinoma cancer; (2) histopathologically confirmed AIS, MIA and IA pulmonary nodules; (3) available CT examination within 1 month before surgery; and (4) the tumor manifesting as GGN on CT with a maximum diameter of (3 mm, 30 mm). The exclusion criteria were: (1) preoperative systemic therapy; (2) lacking CT images before surgery; (3) histopathologically described GGN not identifiable on CT; and (4) artifacts appeared in CT images. We only collected the latest CT examination images of each patient before surgery. The time interval between chest CT examination and operation was 1–30 days (mean, 8.3 days). The institutional review board of two centers approves this retrospective study, and written informed consents were waived from all patients. The details of GGNs in the two centers were depicted as follows.

In the first dataset, we collected 246 GGNs from 229 patients (involving 82 males and 147 females) in Taizhou Municipal Hospital (Zhejiang, China). Among these nodules, 55 GGNs were AIS, 64 GGNs were MIA, and 127 GGNs were IA. All the CT scans were reconstructed by using the standard convolution kernel, and each slice was reconstructed with a matrix 512 × 512 pixels (GE scanner). CT parameters were as follows: 120 kVp tube voltage, and 100–250 mA tube current. The pixel spacing of CT scan ranged from 0.684 to 0.703 mm, and the slice thickness was 1.25 or 5 mm.

The other 127 GGNs were collected from 94 patients (involving 35 males and 59 females) in Fudan University Shanghai Cancer Center (Shanghai, China). In this dataset, 52 AIS GGNs, 34 MIA GGNs, and 41 IA GGNs were involved. The CT examinations were performed with a fixed tube voltage of 120 kVp and a tube current of 200 mA. The pixel spacing of CT image ranged from 0.684 to 0.748 mm, and the slice thickness was 1 or 1.5 mm. Each slice was reconstructed with an image matrix of 512 × 512 pixels.

In order to train and test our proposed schemes, we divided the GGNs into two parts. We used 246 GGNs in the first dataset to build a training and validation dataset to train our scheme. Meanwhile, to evaluate our new scheme performance, we selected the 127 GGNs in the second part to build an independent testing dataset. The details of our dataset were listed in Table 1.

**Table 1:** Demographic characteristics of 323 patients with 373 GGNs in two datasets.

**Characteristic**		**Training and validation dataset (*****N*** **= 246)**			**Testing dataset (*****N*** **= 127)**		
		**Non-IA**	**IA**	***P***	**Non-IA**	**IA**	***P***
		119	127		86	41	
Sex	Male	40	42	0.15	19	16	0.15
	Female	73	74		43	16	
Age (mean ± SD, year)		56.5 ± 11.8	59.7 ± 10.3	0.03	51.8 ± 12.1	58.1 ± 8.6	0.03
Location	RUL	48 (19.5%)	52 (21.1%)	0.64	28 (22.0%)	18 (14.2%)	0.13
	RML	6 (2.4%)	9 (3.7%)		6 (4.7%)	3 (2.4%)	
	RLL	17 (6.9%)	19 (7.7%)		15 (11.8%)	7 (5.5%)	
	LUL	34 (13.8%)	32 (13.0%)		25 (19.7%)	7 (5.5%)	
	LLL	14 (5.7%)	15 (6.1%)		12 (9.4%)	6 (4.7%)	
Diameter (mm)	(3, 10)	72 (29.3%)	42 (17.1%)	0.004	67 (52.8%)	8 (6.3%)	<0.0001
	(10, 20)	39 (15.9%)	68 (27.6%)		19 (15.0%)	22 (17.3%)	
	(20, 30)	8 (3.3%)	17 (6.9%)		0 (0%)	11 (8.7%)	
Type	pGGN	88 (35.8%)	65 (26.4%)	0.0002	78 (61.4%)	18 (14.2%)	<0.0001
	sGGN	31 (12.6%)	62 (25.2%)		8 (6.3%)	23 (18.1%)	

*IA, invasive adenocarcinoma; pGGO, pure ground glass nodule; sGGN, part-solid ground glass nodule*.

### Methods

**Figure 1:**
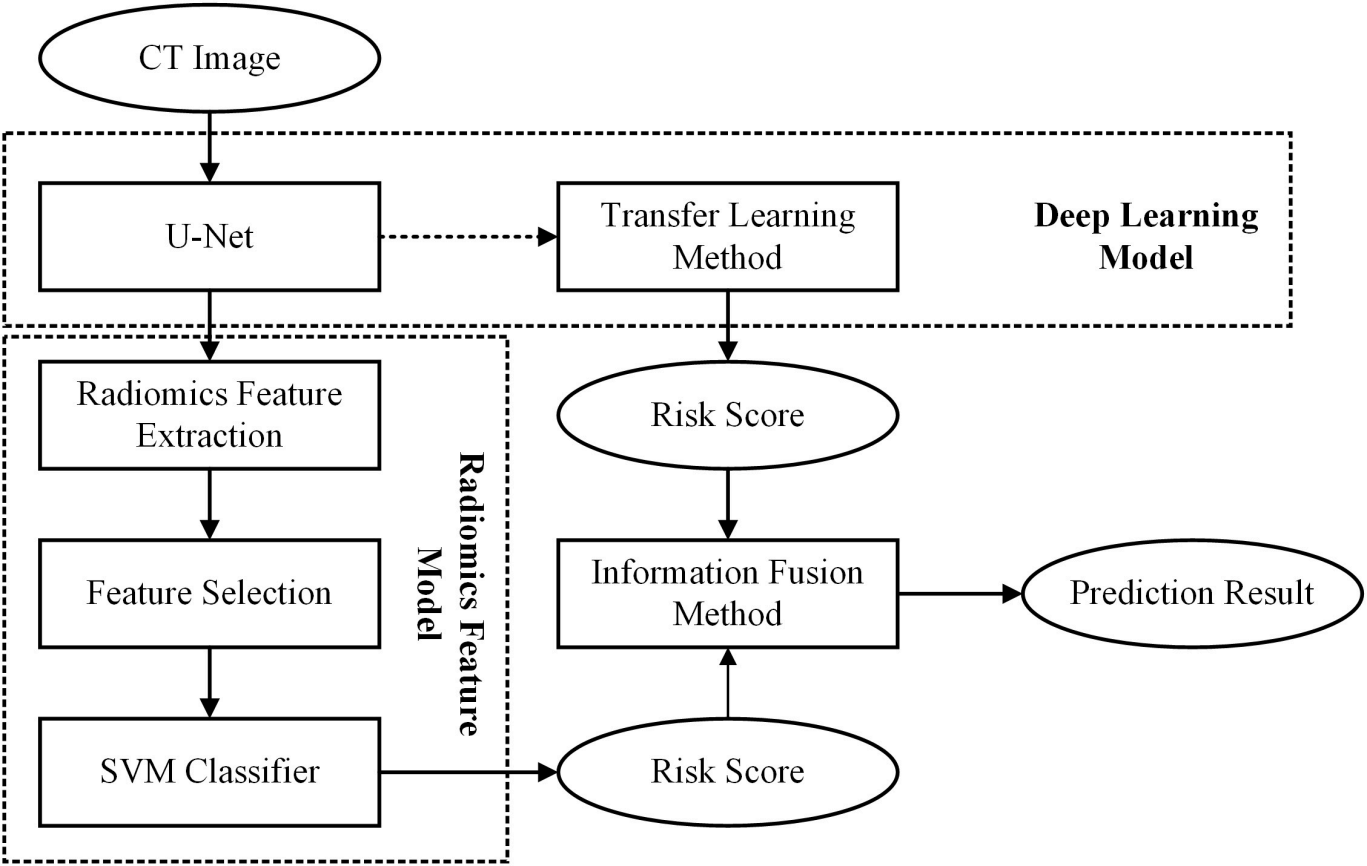
Flowchart of the proposed scheme.

In this study, we first built a DL based model and a radiomics feature based model, respectively. Then, to improve the scheme performance, we used an information-fusion method to fuse the prediction scores of the two schemes. The framework of our proposed scheme was illustrated in Figure 1.

Before building the scheme, we first used a series of preprocessing technique to process the initial CT images. To avoid the biases caused by the variant spacing of CT scans in our dataset, we applied a cubic spline interpolation algorithm to resample CT images to a new spacing of 1 mm × 1 mm × 1 mm. Then, we used an intensity window range of [−1,200, 600] to scale the resampled axial CT images to an intensity range of 0–255. After normalized all the CT images, we cropped the GGN into a 3D cubes with a patch of 64 × 64× 64 mm. During this process, we used the position of GGN center point in Cartesian coordinates drawn by radiologist to locate each GGN in CT image. Last, in order to reduce the computational cost of our model, we normalized the intensity of cropped GGN cubes to an intensity range of 0–1.

**Figure 2:**
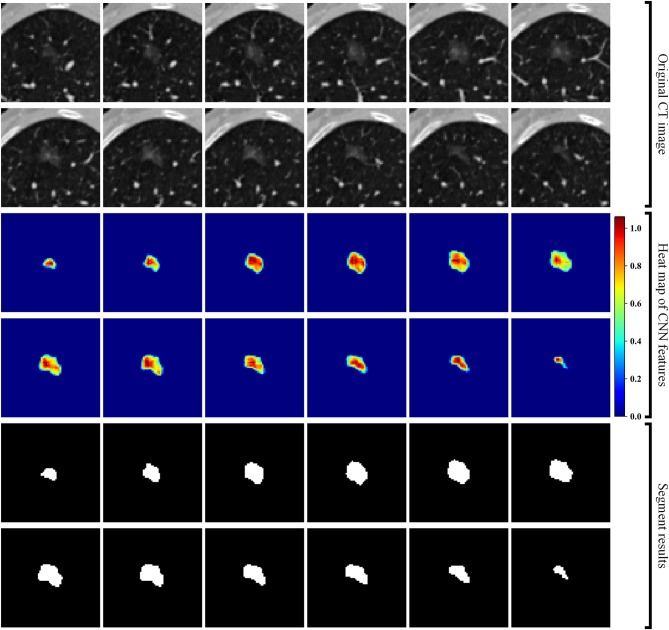
Segmentation results of a GGN. From top to bottom: original CT images, heat map of CNN features, and segment masks of the GGN.

Second, we built a 3D RRCNN based on U-Net model to segment the GNNs in CT images. The architecture of our segmentation DL model were showed in Figure 2. The inputs of 3D RRCNN model were our cropped GGN patches, and the outputs were the segmented 3D masks. For each layer of the 3D RRCNN, we used a RRCNN block with a 3 × 3 × 3 convolutional layer, a batch normalization layer and a standard rectified linear unit (ReLU). In each convolutional layer, we also embedded a residual unit and a recurrent unit into the block (21). To build the segmentation model, we used the 257 GGNs in the lung image database consortium and image database resource initiative (LIDC-IDRI) to train our proposed RRCNN model (22). Four radiologists delineated the boundaries of nodules in LIDC-IDRI database. We used the boundary voted by three or more radiologists as the “ground-truth” of each nodule. To generate the training GGNs for RRCNN model, we applied some data augmentation techniques (i.e., rotation of image by 90° increments, left-right flipping, up-down flipping) to augment the dataset. Moreover, we applied the Dice similarity coefficient (DSC) of nodule to define the loss function of our segmentation model (23). Figure 2 shows an example of GGN segmentation results.

**Figure 3:**
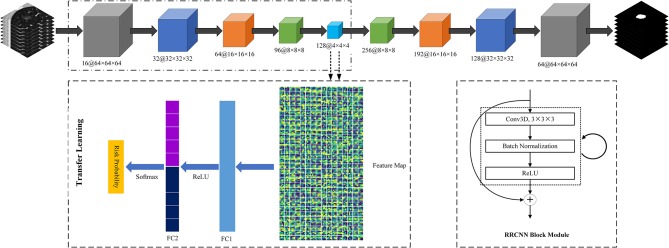
The architectures of Recurrent Residual Convolutional Neural Network (RRCNN) based on U-Net model and the transfer learning method based risk prediction model.

Third, we used a transfer learning method to build a DL based invasiveness risk prediction model. In this model, we fixed the parameters in CNN-pooling processes of the segmentation model. To build a classification model, we added two fully connected (FC) layers into the DL model, and used deep features generated by the CNN-pooling layers of segmentation model to feed into the FC layers. Then, we used the GGNs in our training and validation dataset to fine-tune our classification CNN model. In this process, we selected the cross entropy to calculate the loss, and used an Adam optimizer with a weight decay of 1e-4 to update the parameters. Figure 3 shows the architectures of our proposed DL model.

Fourth, we built a radiomics feature analysis model to classify between non-IA and IA GGNs. For each CT scan in our dataset, we used the RRCNN model to segment 3D GGNs. Then, we computed 1,218 radiomics features to quantify each GGN. These imaging features involved: 430 LoG features, 688 wavelet features, 18 histogram features, 14 shape features, and 68 texture features. The LoG features and wavelet features were computed by using the Laplacian of Gaussian (LoG) filter and wavelet filter to filter the initial image, respectively. The LoG image was obtained by convolving the original image with the second derivative of a Gaussian kernel. Five sigma values including 1, 2, 3, 4, and 5 were used to calculate the LoG features. In Among the 68 texture features, 22 were gray level co-occurrence matrix texture features (GLCM), 14 were gray level dependence matrix texture features (GLDM), 16 were gray level run length matrix texture features (GLRLM), and 16 were gray level size zone matrix texture features (GLSZM). After extracting the radiomics features, we scaled each feature to [0, 1] by using a feature normalization technique. To reduce the dimensionality of initial features, we applied the univariate feature selection method with ANOVA *F*-value to select the best features and remove the redundant features (24). After feature selection processing, we used these selected imaging features to train a support vector machine (SVM) classifier and build a radiomics feature based model.

Finally, we used an information-fusion method to fuse the prediction scores of two classification models. In brief, the information-fusion strategies includes the maximum, minimum, and weighting average fusion. For maximum and minimum strategy, we compared two prediction scores of each GGN, and selected the maximum or minimum value as the fusion prediction score. For weighting average strategy, we systematically increased the weighting factor of prediction score generated by DL based scheme from 0.1 to 0.9 (or 0.9–0.1 for the prediction score generated by radiomics feature based scheme) to compute the fusion prediction score. A similar method was applied in our previously reported literature (25).

### Performance Evaluation

After obtaining the prediction scores, we generated the receiver operating characteristic (ROC) curves and computed the area under a ROC curve to evaluate the performance of our proposed models. In order to compare the new scheme performance with radiologists, we conducted an observer study by testing on an independent testing dataset. Two radiologists (a junior radiologist: Wen Hao with 5-years experience; a senior radiologist: Shengping Wang with 14-years experience in CT interpretation) were independently to diagnose all the GGNs in testing dataset by blinding to the histopathologic results and clinical data. Since two radiologists only provided a binary result for each case, we calculated some additional metrics to assess and compare the prediction performance. The evaluation indexes were accuracy (ACC), F1 score, weighted average F1 score, and Matthews correlation coefficient $\left(\text{MCC}=\frac{\text{TP}\times \text{TN}-\text{FP}\times \text{FN}}{\sqrt{\left(\text{TP}+\text{FP}\right)\left(\text{TP}+\text{FN}\right)\left(\text{TN}+\text{FP}\right)\left(\text{TN}+\text{FN}\right)}}\right)$, respectively. The equation of F1 score was defined as follows.

$$\text{F}1=\frac{2\times \text{Precision}\times \text{Recall}}{\text{Precision}+\text{Recall}}$$

where TP, FP, TN, FN denoted true positive, false positive, true negative, and false negative, respectively. Precision denoted the precision value $\left(\text{Precision}=\frac{\text{TP}}{\text{TP}+\text{FP}}\right)$, and Recall denoted the recall value $\left(\text{Recall}=\frac{\text{TP}}{\text{TP}+\text{FP}}\right)$.

In this study, we implemented the above model building and performance evaluation processes on the Python 3.6 by using a computer with Intel Core i7-8700 CPU 3.2 GHz × 2, 16 GB RAM and a NVIDIA GeForce GTX 1,070 graphics processing unit. To build the DL and radiomics feature based scheme, we applied some publicly available Python packages, i.e., SimpleITK, pyradiomics (26), Pytorch, scikit-learn, scikit-feature, scipy. We used the default configuration of performance evaluation functions. Thus, the scheme performance can be easily compared and evaluated in future studies.

All the codes of our proposed models were open source available at https://github.com/GongJingUSST/DL_Radiomics_Fusion.

## Results

**Figure 4:**
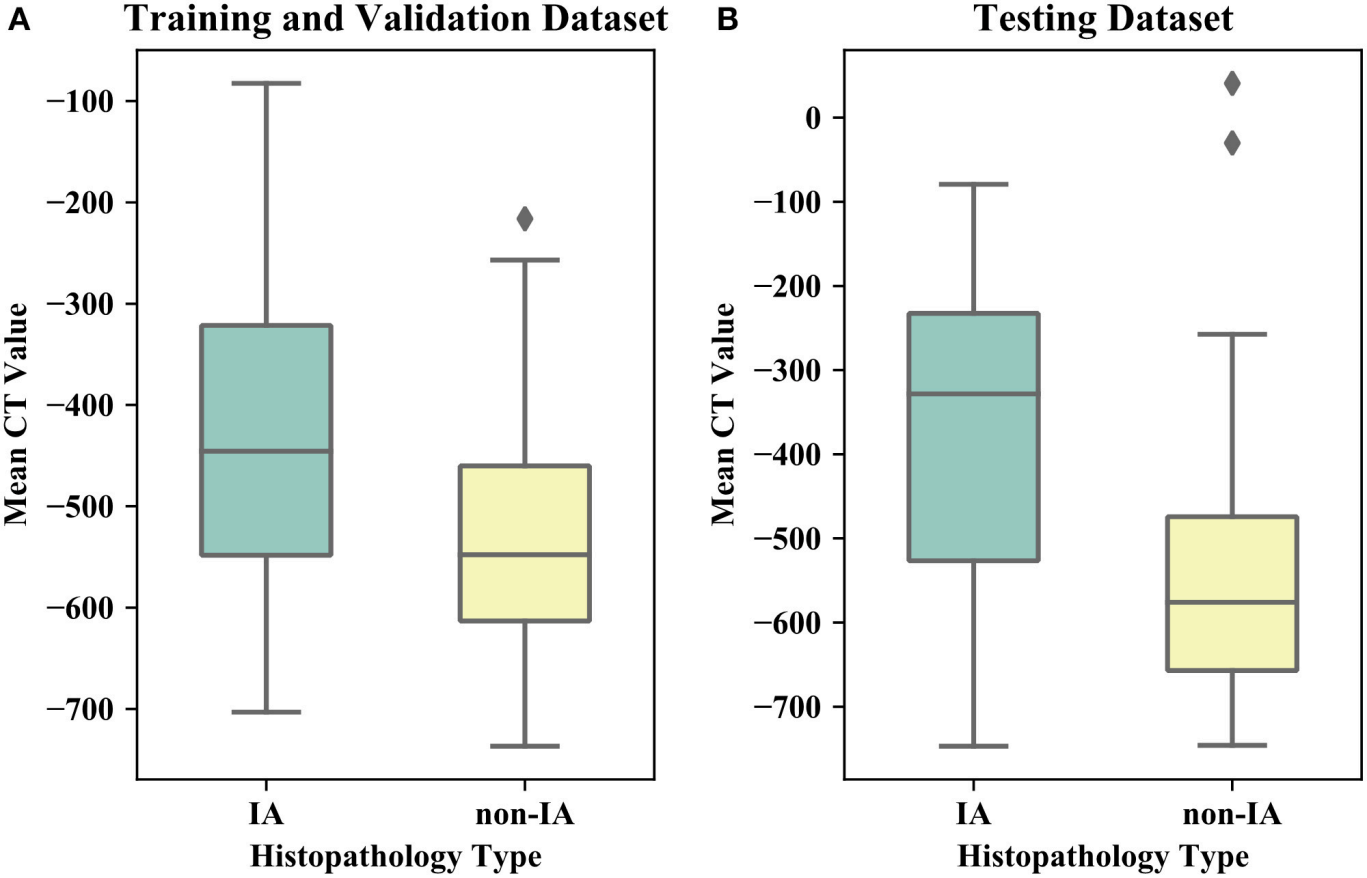
Boxplots of the mean CT value of IA and non-IA GGNs in our dataset. **(A)** Illustrates boxplot of the training and validation dataset. **(B)** Shows boxplot of the testing dataset.

Table 1 listed the detailed demographic characteristics of the patients in two datasets. A total of 323 patients [117 (36.2%) males, and 206 (63.8%) females, *P* > 0.05] with 373 GGNs were involved in our dataset. Among these GGNs, 107 were AIS (28.7%), 98 were MIA (26.3%), and 168 were IA (45%). Of all 373 GGNs, 228 (61.1%) were located in right lobe, and 145 (38.9%) were located in left lobe (*P* > 0.05). In the dataset, the diameters of 189 (50.7%) GGNs were smaller than 10 mm, the diameters of 148 (39.7%) GGNs were in a range of (10 mm, 20 mm), and the diameters of 36 (9.6%) GGNs were larger than 20 mm (*P* < 0.05). Of 373 GGNs, 249 nodules (66.8%) showed pure GGNs without solid components, and 124 nodules (33.2%) showed part-solid GGNs on CT images. Figure 4 illustrates the boxplots of GGN mean CT values in training and testing dataset. In training and validation dataset, the mean CT value of IA and non-IA GGNs were −439 ± 138 and −533 ± 116, respectively. Meanwhile, in the testing dataset, the mean CT value of IA and non-IA were −381 ± 182 and −553 ± 142.

**Figure 5:**
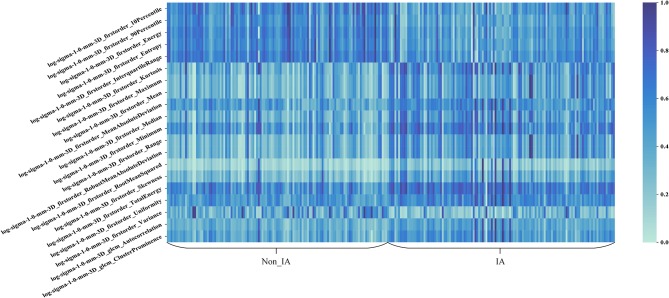
Heat map of the 20 imaging features selected in the radiomics based model.

Figure 5 shows the heat map of the 20 selected imaging features in the radiomics feature based scheme. In Figure 5, these 20 imaging features selected from the initial feature pool were LoG image based features. It can be seen that LoG features play an important role in building the radiomics feature based classification model. Most of the selected imaging features have a different distribution between non-IA and IA GGNs. It indicated that most of these selected features have a potential to differ non-IA from IA GGNs.

Table 2 listed the AUC values and the corresponding 95% confidence interval (CI) of the models proposed in this study. Testing on the independent testing dataset, the DL based scheme and radiomics feature based scheme yielded an AUC value of 0.83 ± 0.05 and 0.87 ± 0.04, respectively. When we applied the information-fusion method, the scheme performance changed with the different fusion strategy. By using a maximum fusion strategy, our scheme yielded a highest AUC value of 0.90 ± 0.03. Comparing with the performance generated individually, the fusion scheme significantly improved the scheme performance (*P* < 0.05). Meanwhile, there is no significant difference between DL based scheme and radiomics feature based scheme (*P* = 0.09).

**Table 2:** AUC values and the corresponding 95% CI generated by different methods with 127 GGNs in testing dataset.

**Method**	**AUC**	**95% CI**
Deep learning based scheme	0.83 ± 0.05	[0.75, 0.90]
Radiomics feature based scheme	0.87 ± 0.04	[0.80, 0.93]
Minimum	0.83 ± 0.05	[0.75, 0.90]
Maximum	0.90 ± 0.03	[0.84, 0.95]
0.1 × Radiomics^a^+0.9 × DL^b^	0.85 ± 0.04	[0.77, 0.91]
0.2 × Radiomics+0.8 × DL	0.86 ± 0.04	[0.78, 0.92]
0.3 × Radiomics+0.7 × DL	0.87 ± 0.04	[0.80, 0.93]
0.4 × Radiomics+0.6 × DL	0.88 ± 0.04	[0.81, 0.94]
0.5 × Radiomics+0.5 × DL	0.89 ± 0.04	[0.83, 0.95]
0.6 × Radiomics+0.4 × DL	0.90 ± 0.04	[0.83, 0.95]
0.7 × Radiomics+0.3 × DL	0.90 ± 0.04	[0.83, 0.90]
0.8 × Radiomics+0.2 × DL	0.90 ± 0.04	[0.83, 0.88]
0.9 × Radiomics+0.1 × DL	0.89 ± 0.03	[0.83, 0.94]

a *Radiomics: prediction scores generated by radiomics feature based scheme*.

b *DL: prediction scores generated by deep learning based scheme*.

**Figure 6:**
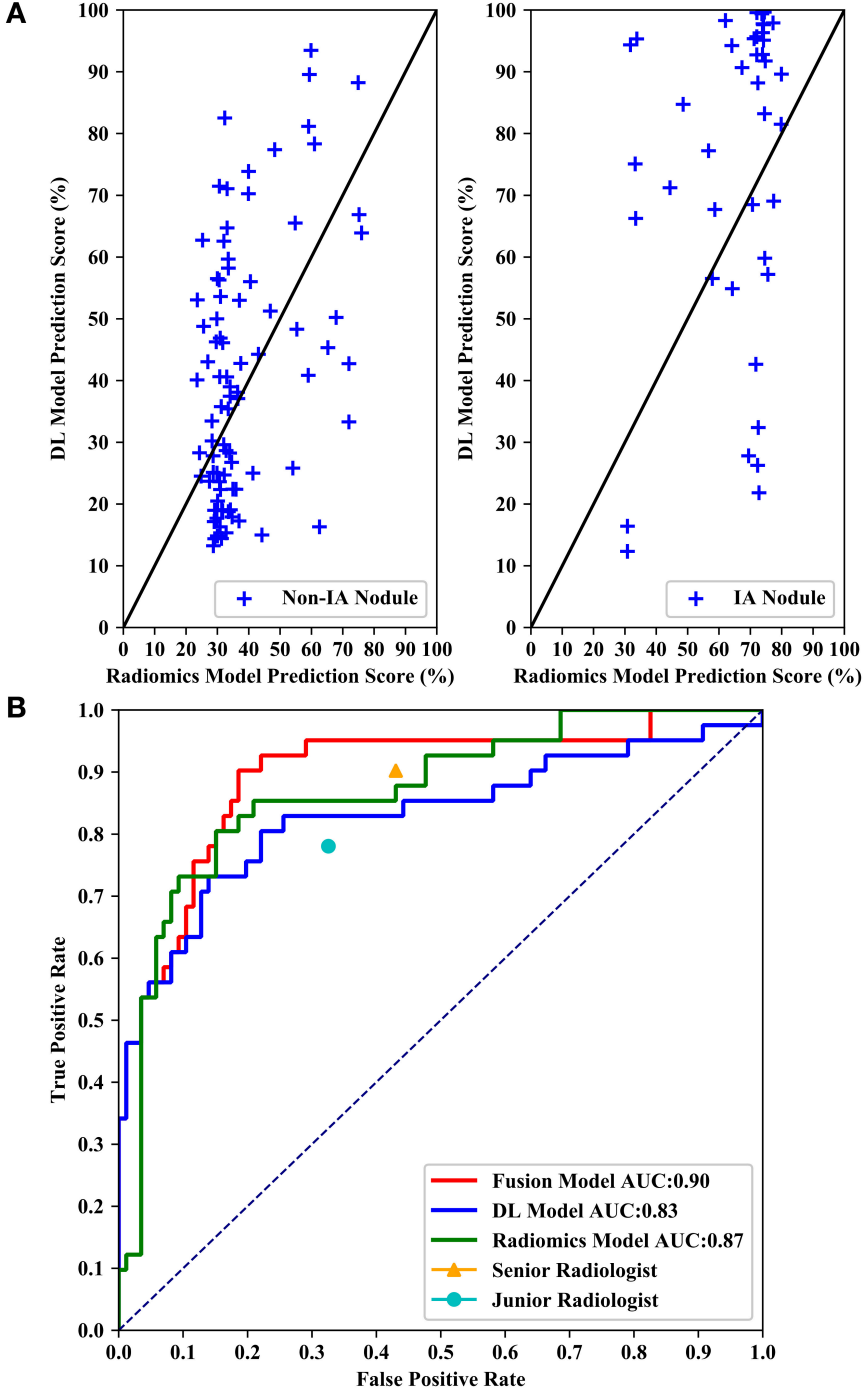
Performance comparisons of three models and radiologists. **(A)** Shows scatter plots of prediction score distributions of non-IA and IA nodules. Left to right: prediction scores generated by DL and radiomics models for non-IA and IA nodules in testing dataset, respectively. **(B)** Shows ROC curves of the three models and the prediction scores of two radiologists.

Figure 6 shows performance comparisons of three models and radiologists. Figure 6A shows scatter plot of prediction score distributions of non-IA and IA nodules, and Figure 6B shows ROC curves of the three models and the prediction scores of two radiologists. Figure 6A showed that a large number of prediction scores generated by DL and radiomics based models were scattered and inconsistent in both non-IA and IA nodules. It indicated DL model and radiomics model might provide different information in classifying between non-IA and IA nodules. ROC curves also showed the trend that fusing the scores of DL based scheme and radiomics feature based scheme can improved the scheme performance. In a comparison with two radiologists, the fusion scheme yielded higher performance. In order to further compare the fusion scheme performance with two radiologists, Table 3 illustrated and compared the accuracy, F1 score, weighted average F1 score, and Matthews correlation coefficient of each scheme. Evaluating the results showed in Table 3, our fusion scheme yielded higher performance than two radiologists in terms of each index. It indicated that our CADx scheme matched or even outperformed radiologist in classifying between non-IA an IA GGNs. To test the interrater reliability of the results of two radiologists, we also calculated the Cohen's kappa value to measure their agreement (27). The Cohen's kappa value of two radiologists was 0.6. It indicated that two radiologists had a moderate agreement in predicting the invasiveness risk of GGN.

**Table 3:** The comparison of classification performance tested on 127 GGNs in independent testing dataset, in terms of accuracy (ACC), F1 score, weighted average F1 score, and Matthews correlation coefficient (MCC), respectively.

	**ACC (%)**	**F1 (%)**	**F1_**weighted**_ (%)**	**MCC (%)**
Senior radiologist	67.7	64.3	68.5	44.8
Junior radiologist	70.9	63.4	71.8	42.6
Our fusion model	80.3	75.2	80.9	62.8

## Discussion

In this study, we developed a CT image based CADx scheme to classify between non-IA and IA GGNs by fusing DL and radiomics features. Our study has a number of characteristics. First, we built an AI model to classify between non-IA and IA GGNs by fusing DL and radiomics features. Since DL based scheme and radiomics feature based scheme used different imaging features to decode the phenotypes of GGN, our fusion model integrated these quantitative and deep features to character the CT features of tumor. Comparing with model built with DL and radiomics features individually, the fusion model has improved the scheme performance significantly (i.e., results showed in Table 2 and Figure 6). It showed that deep feature and radiomics feature may provide complementary information in predicting the invasiveness risk of GGN. To build a robust model, we used the surgery histopathological confirmed GGNs from two centers to train and test the classification scheme. In order to evaluate the performance of our scheme, we compared the scheme prediction scores with two radiologists by testing on an independent dataset. Comparing with two radiologists, our new scheme yielded higher performance in classifying between non-IA and IA GGNs (i.e., results showed in Figure 6 and Table 3). Meanwhile, comparing with previously reported studies (15, 19, 28), our study can yield a rather high classification performance by using a limited dataset (i.e., results showed in Table 4). If the robustness of our model was confirmed with more diverse and larger dataset in future studies, the proposed AI scheme would have a high impact on assisting radiologists in their clinical diagnosis of GGNs.

**Table 4:** Comparison of dataset, methods, and AUC values reported in different studies.

**Work**	**Dataset**	**Method**	**AUC**
Wang et al. (19)	1,545 nodules	Deep learning	0.892
Zhao et al. (15)	651 nodules	Deep learning	0.880
Gong et al. (28)	828 nodules	Deep learning	0.92 ± 0.03
Our study	373 nodules	Fusion of deep learning and radiomics	0.90 ± 0.03

Second, we applied a transfer learning method to build a DL based scheme by training with a limited dataset. Since the DL based scheme was a data-driven model, we should train and build a DL model with a large dataset. To address this issue, we proposed a RRCNN model to segment GGNs, and then used a transfer learning method to fine-tune the segmentation DL model. In this process, our classification DL model shared the same deep features with the segmentation model. As the training images of two model was same, it was easily to transfer the segmentation model to classification task. In a comparison with radiomics feature based model, the DL based scheme yielded equivalent performance (*P* > 0.05). It demonstrated that transferring segmentation DL model to classification task was feasible. Thus, our new scheme may provide a new way to build a DL based classification model with limited dataset.

Third, we built a radiomics feature based scheme to predict the invasiveness risk of GGN. To quantify the imaging phonotypes of GGN, we initially computed 1,218 radiomics features. To remove the redundant imaging features, we applied a univariate feature selection method to select the robust features. Most of the selected imaging features were LoG image based features. It showed that LoG features were essential for classifying between non-IA and IA GGNs. By observing the heat map of 20 selected image features, we found that those features had a different distributions in non-IA and IA group. It indicated that these selected imaging features had a potential to classify between non-IA and IA GGNs.

Fourth, in order to evaluate the performance of our proposed scheme, we conducted an observer study by comparing with two radiologists. Senior radiologist obtained higher sensitivity (90.2 vs. 78.1%) and false positive rate (43.0 vs. 32.6%) in distinguishing between IA and non-IA GGNs. It indicated that senior radiologist was more sensitive to the positive GGNs (i.e., IA GGNs). Meanwhile, the accuracy of senior radiologist was lower than that of junior radiologist. Since the number of non-IA GGNs is larger than that of IA GGNs in our testing dataset, it indicated that the number of negative GGNs (i.e., non-IA GGNs) miscategorized into IA class by senior radiologist was larger. Thus, senior radiologist paid more attention to IA GGNs than non-IA GGNs. Two radiologists had a moderate agreement on diagnosing the invasiveness risk of GGNs. By validating on an independent testing dataset, our AI scheme outperformed two radiologists in classifying between non-IA and IA GGNs (i.e., results showed in Table 3 and Figure 6). It demonstrated that CT image based AI scheme was an effective tool to distinguish between non-IA and IA GGNs. Due to the different ways of surgical management for GGNs with different subtypes of lung adenocarcinoma, our AI scheme may have a potential to assist both radiologists and thoracic surgeons in their decision-making.

Despite of the promising results, this study also had several limitations. First, our dataset was small, and only a total of 373 GGNs were involved in this study. The diversity of GGNs in our dataset cannot sufficiently represent the general GGN population in clinical practice. Since the DL model was data-driven, it may be under-fitting due to lack of training dataset. Thus, large diverse dataset and cross-validation method should be used to validate the reproducibility and generalization of our scheme. Due to the different scanning parameters, the tube current, pixel spacing, and slice thickness of CT image was variety. Whether and how these scanning parameters affect the scheme performance have not been investigated in this study (29).

Second, we only extracted and investigated two type CT image features of lung adenocarcinoma namely, DL image feature and radiomics feature, respectively. Although the scheme performance has been improved by fusing two types of imaging features, CT image features cannot decode the whole phenotypes of lung adenocarcinoma tumor. The clinical data, such as smoking history, family history, carcinogenic exposure history, chronic obstructive pulmonary disease, emphysema, interstitial lung disease, etc., may also provide useful classification information. In future studies, we should also apply and combine other types of features (i.e., clinical information, tumor biomarkers, gene feature) to improve the scheme performance (30).

Third, to improve the scheme performance, we only applied a simple information-fusion method to fuse the prediction scores of DL and radiomics based scheme. Due to the limited dataset, our proposed DL scheme and radiomics model may be over-fitting during training process. By applying different weights to the prediction scores of two models, fusion model can weak the over-fitted model's impacts. The over-fitting can be alleviated to some degree by fusing the prediction scores generated by two models. Although the scheme performance has been improved, it may not be the optimal way to combine two types of image features. Thus, we should investigate and develop new fusion methods to fuse the different types of features in future studies. The weak interpretation of DL based scheme is also a limitation of this study. In addition, we used the positions delineated by radiologist to crop GGN patches and generate the training and testing images. The human intervention may also affect the scheme performance.

Last, in our observer study, two radiologists read CT images with time and information constraints, which is different from real clinical situation. The insufficient diagnosis time and clinical information may result in the low performance of two radiologists. Moreover, this is an only technique development study, and we need to conduct rigorous and valid clinical evaluation before applying the proposed scheme into clinical practice.

## Conclusion

In this study, we developed an AI scheme to classify between non-IA and IA GGNs in CT images. To improve the scheme performance, we fused the prediction scores generated by DL based scheme and radiomics feature based scheme, respectively. The results shows that fusion of DL and radiomics features can significantly improve the scheme performance. Comparing with two radiologists, our new scheme achieves higher performance. It demonstrates (1) fusing DL and radiomics features can improve the classification performance in distinguishing between non-IA and IA, (2) we can build classification DL model with the limited dataset by transferring segmentation task to classification task, (3) AI scheme matches or even outperform radiologists in predicting invasiveness risk of GGNs. Therefore, to improve the diagnosis performance of GGNs, one should focus on exploring and computing robust imaging features, and developing optimal method to fuse different types of features.

## Data Availability Statement

The datasets generated for this study are available on request to the corresponding author.

## Ethics Statement

The studies involving human participants were reviewed and approved by Municipal Hospital Affiliated to Medical School of Taizhou University and Fudan University Shanghai Cancer Center. The ethics committee waived the requirement of written informed consent for participation.

## Author's Note

In this study, we investigate and develop CT image based artificial intelligence (AI) schemes to predict the invasiveness risk of lung adenocarcinomas, and incorporate deep learning (DL) and radiomics features to improve the prediction performance. The results show that (1) fusing DL and radiomics features can improve the classification performance in distinguishing between non-IA and IA, (2) we can build classification DL model with limited dataset by transferring segmentation task to classification task, (3) AI scheme matches or even outperform radiologists in predicting invasiveness risk of GGNs.

## Author Contributions

JG and SW designed this study. JG, XX, TY, YL, and SW performed the search and collected data. JG performed data analysis and wrote the manuscript. WH and SW independently diagnosed all the GGNs in testing dataset. All authors reviewed the manuscript.

### Conflict of Interest

The authors declare that the research was conducted in the absence of any commercial or financial relationships that could be construed as a potential conflict of interest.
